# Fibroblast Growth Factor 21 Analogues Improve Fibrosis in Metabolic Dysfunction–Associated Steatohepatitis: An Updated Systematic Review and Meta‐Analysis

**DOI:** 10.1155/ijh/7391450

**Published:** 2026-06-03

**Authors:** Muhammad Naqash, Abdullah Tariq, Shazma Shayan, Sadaf Manzoor Sargani, Muhammad Asad Raza, Nasar Nasrullah Khan, Aiman Balouch, Rana Muhammad Usama, Khabab Abbasher Hussien Mohamed Ahmed

**Affiliations:** ^1^ Hull University Teaching Hospitals NHS Trust, Hull, UK; ^2^ Nottingham University Hospital NHS Trust, Nottingham, UK, nhs.uk; ^3^ Hull University Teaching Hospitals NHS Foundation Trust, Hull, UK; ^4^ Northern Lincolnshire and Goole NHS Foundation Trust, Scunthorpe, UK, nhs.uk; ^5^ Lahore General Hospital, Lahore, Pakistan; ^6^ Faculty of Medicine, University of Khartoum, Khartoum, Sudan, uofk.edu

**Keywords:** fibroblast growth Factor 21 (FGF21), liver fibrosis, liver-derived hormone, metabolic dysfunction–associated steatohepatitis (MASH), nonalcoholic fatty liver disease (NAFLD)

## Abstract

**Background:**

Metabolic dysfunction–associated steatohepatitis (MASH) (formerly nonalcoholic steatohepatitis, MASH) is a growing cause of liver morbidity worldwide. Although therapeutic options for MASH fibrosis have expanded, including the approval of resmetirom for noncirrhotic MASH with F2–F3 fibrosis, additional therapies with antifibrotic efficacy remain needed. Fibroblast growth Factor 21 (FGF21) is a liver‐derived hormone that regulates lipid metabolism, insulin sensitivity, and energy balance, and long‐acting FGF21 analogues have shown promise in Phase 2 trials. We performed a systematic review and meta‐analysis to quantify the efficacy and safety of FGF21 analogues in improving liver fibrosis in MASH.

**Methods:**

We searched PubMed, Scopus, and the Cochrane Library (through February 2026) for randomized controlled trials (RCTs) comparing an FGF21 analogue versus placebo in adults with biopsy‐confirmed MASH. Primary outcomes were ≥ 1‐stage histological fibrosis improvement without MASH worsening. Secondary outcomes included changes in hepatic fat, liver enzymes, and key metabolic parameters. Data were pooled using random‐effects models, calculating pooled risk ratios (RRs) or standardized mean differences (SMDs) with 95% confidence intervals (CI).

**Results:**

We identified 10 RCTs (total: 1113 patients with F1–F4 fibrosis) meeting inclusion criteria. FGF21 analogue treatment significantly increased the likelihood of achieving ≥ 1‐stage fibrosis improvement (RR: 2.25, CI: 1.25–4.03) compared with placebo. FGF21 analogues also produced larger reductions in hepatic fat content, liver stiffness, and fibrosis biomarkers than placebo. The incidence of serious adverse events was similar between groups. The most reported treatment‐related side effects were gastrointestinal, especially nausea and diarrhea, and local injection reactions; these were generally mild and did not significantly limit therapy.

**Conclusions:**

FGF21 analogue therapy is associated with significantly greater histological fibrosis improvement in patients with MASH and appears to be well tolerated. These findings suggest that FGF21 analogues may become a valuable pharmacotherapy for MASH, but confirmatory larger trials and long‐term data are needed.

## 1. Introduction

Global liver morbidity is increasingly driven by metabolic dysfunction–associated steatohepatitis (MASH). Although metabolic dysfunction–associated steatotic liver disease (MASLD) affects nearly a third of the population, [1] a significant subgroup progresses to steatohepatitis and fibrosis, representing a critical area of unmet clinical need. [2] A substantial subset of these individuals (on the order of 20%–30%) develop progressive steatohepatitis and fibrosis. By current nomenclature, NAFLD has been redefined as metabolic dysfunction–associated steatotic liver disease (MASLD), and MASH replaces MASH in this framework. [3] MASH is characterized by hepatic steatosis, inflammation, and hepatocellular injury, often in the context of obesity, insulin resistance, Type 2 diabetes, hypertension, and dyslipidemia. Over time, unchecked MASH can lead to cirrhosis, hepatocellular carcinoma, liver failure, and substantial morbidity. [4] Despite the rising burden, therapeutic options are limited. Lifestyle modification and weight loss remain first‐line, and only resmetirom has been approved for Stages F2–F3 MASH fibrosis. [5] In this context, novel pharmacotherapies are urgently sought.

Fibroblast growth Factor 21 (FGF21) is a liver‐derived hormone that has attracted attention in metabolic disease. [6] FGF21 regulates lipid and glucose metabolism, enhances insulin sensitivity, promotes weight loss, and exerts anti‐inflammatory and antifibrotic actions in preclinical models. [7] In animal studies, FGF21 analogues ameliorate hepatic steatosis and fibrogenesis. [8] Human genetic and experimental data suggest that higher FGF21 activity may counteract the drivers of MASH. [8, 9] Accordingly, several long‐acting FGF21 analogue drugs (such as the Fc‐fusion efruxifermin, the pegylated pegbelfermin, and the glycopegylated pegozafermin) have been developed and tested in Phase 2 trials of biopsy‐proven MASH. [10] These trials have generally shown impressive reductions in liver fat and improvements in metabolic parameters, and some have reported improvements in histology. For example, in a Phase 2b trial of pegozafermin (ENLIVEN), [11] roughly a quarter of patients on active drug achieved ≥ 1‐stage fibrosis reduction by Week 24, versus only ~7% with placebo. Efruxifermin trial (HARMONY) [12] has likewise shown reductions in hepatic fat and transaminases. Safety signals have been favorable, with mostly mild gastrointestinal effects.

Despite encouraging individual trials, it remains unclear to what extent these histological effects are robust and consistent across studies. Meta‐analysis can provide a more precise estimate by pooling all available randomized data. Previous reviews of FGF21 analogues have primarily focused on metabolic outcomes; more recent meta‐analyses have begun to quantify effects on histology and fibrosis, but the literature is rapidly evolving. Additionally, other analyses have suggested uncertainty, particularly regarding MASH resolution endpoints. Furthermore, most published meta‐analyses predate the latest trials and use the older MASH terminology rather than MASH.

Given this landscape, we conducted a comprehensive systematic review and meta‐analysis of all randomized trials of FGF21 analogue therapy in patients with biopsy‐confirmed MASH. We focused on the histological endpoints defined by regulatory guidance (fibrosis improvement and MASH resolution) while also summarizing changes in liver fat, serologic biomarkers, and safety outcomes. Our goal was to clarify the efficacy of the FGF21 drug class in improving fibrosis and other clinically relevant outcomes in MASH, and to evaluate consistency across studies.

## 2. Methods

We conducted this systematic review per PRISMA guidelines, [13] performing a comprehensive literature search and rigorous data synthesis. A search strategy was developed to identify randomized controlled trials (RCTs) of FGF21 analogues in adults with MASH (biopsy‐proven) published through February 2026. We searched PubMed, Scopus, and Cochrane Central using terms related to “FGF21” or specific analogue names (e.g., efruxifermin, pegbelfermin, and pegozafermin) combined with “steatohepatitis,” “MASH,” “MASH,” and “fibrosis.” Reference lists of relevant articles were also screened. Table S1 highlights the detailed search string used in each database. The study protocol was registered with PROSPERO with ID No. CRD420251017463.

### 2.1. Study Selection

A dual‐review process was employed wherein two investigators independently evaluated records against eligibility requirements, first at the title/abstract level and subsequently via full‐text review. Any discordant decisions regarding study inclusion were settled through discussion to reach a unanimous decision. We included parallel‐arm RCTs comparing any long‐acting FGF21 analogue (any dose, any route) versus placebo, in adults with biopsy‐confirmed MASH (MASH) of any fibrosis stage. Trials must have reported at least one histological outcome (fibrosis or steatohepatitis). For multiarm trials with more than one eligible FGF21 analogue dose arm and a shared placebo group, we used a standardized approach to avoid double counting of placebo participants. All eligible active FGF21 arms were combined into a single active‐treatment group and compared with the shared placebo group for the primary analysis. For dichotomous outcomes, event counts and total sample sizes were summed across eligible active arms. For continuous outcomes, active‐arm means and standard deviations were combined using standard Cochrane methods based on sample size, mean, and variance. No active treatment arm was preferentially selected over another for the primary analysis. Exclusion criteria were nonrandomized studies, trials without a placebo comparator, Phase 1 studies without histology, or studies in pediatric or non‐MASH populations.

### 2.2. Data Extraction and Quality Assessment

From each eligible trial, we extracted trial and patient characteristics (sample size, agent and dose, duration, baseline fibrosis stage, comorbidities, etc.). Two reviewers independently extracted data on outcomes. The coprimary outcomes were (1) ≥ 1‐stage improvement in liver fibrosis (on the MASH Clinical Research Network scale) without worsening of MASH, and (2) resolution of steatohepatitis (defined as no ballooning and at most mild inflammation) without worsening fibrosis. Secondary outcomes included percent relative change in hepatic fat fraction (MRI‐PDFF), changes in fibrosis biomarkers (e.g., PRO‐C3 and ELF score), liver enzymes (alanine aminotransferase [ALT] and aspartate aminotransferase [AST]), and metabolic measures (HDL‐C and LDL‐C). We also recorded safety outcomes, including incidence of serious adverse events and common treatment‐related side effects. For efficacy outcomes, we preferentially extracted intention‐to‐treat or modified intention‐to‐treat data as reported by the original trialists; when unavailable, the closest prespecified biopsy‐evaluable efficacy population was used. For safety outcomes, we extracted data from the safety population, defined as all participants receiving at least one dose of study treatment. For trials with multiple follow‐up reports, we preferentially extracted histologic efficacy data from the earliest biopsy assessment window most comparable with the primary efficacy assessment time points across the included studies, rather than from the longest available follow‐up. This approach was used to reduce clinical heterogeneity related to treatment duration and endpoint timing. To evaluate the methodological integrity of the selected trials, we utilized the Cochrane Collaboration′s RoB 2 instrument. [14] The certainty of evidence for each key outcome was assessed using the GRADE approach across the domains of risk of bias, inconsistency, indirectness, imprecision, and publication bias. Because all included studies were RCT, certainty started at high and was downgraded when concerns were identified in one or more domains (Table S2).

### 2.3. Statistical Analysis

Binary endpoints, including fibrosis improvement and adverse events, were synthesized using random‐effects models. We reported these outcomes as risk ratios (RRs) with corresponding 95% confidence intervals (CI), utilizing the Mantel–Haenszel method. Continuous outcomes (e.g., change in hepatic fat fraction [HFF] and ALT) were pooled as weighted mean differences or standardized mean differences (SMDs) with 95% CI. When continuous outcomes were reported as medians with interquartile ranges or ranges, means and standard deviations were estimated using the methods described by Wan et al. [15] when the distributional assumptions were considered appropriate for quantitative synthesis. When conversion was not appropriate because of skewed data, incomplete reporting, or incompatible outcome definitions, the outcome was summarized narratively and was not pooled. For studies reporting least squares mean changes with standard errors, standard deviations were derived using the formula SD = SE × √*n*. When 95% CI were reported around least squares mean changes, the standard error was calculated as SE = (upper confidence limit − lower confidence limit)/(2 × 1.96) and then converted to standard deviation when required for pooling. For multiarm studies, combined means and standard deviations were calculated using standard formulas that incorporate the sample size, mean, and variance from each active arm. To evaluate inconsistency across studies, we calculated the *I* ^2^ statistic and Cochran′s *Q*. [16] An *I* ^2^ value exceeding 60% was considered indicative of high heterogeneity, whereas values below 30% represented low heterogeneity. Forest plots were generated for key outcomes. We performed sensitivity analyses, excluding any outlier study and assessed the robustness of findings. A funnel plot evaluated publication bias if ≥ 10 studies were available. [16] All analyses were performed in Review Manager 5.4.

## 3. Results

### 3.1. Study Selection and Characteristics

**Figure 1:**
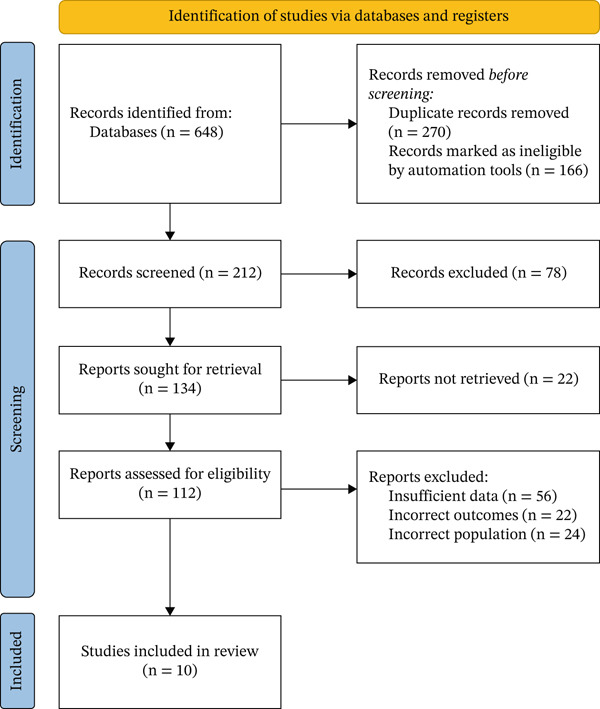
PRISMA flowchart showing the study selection process.

Our search yielded 590 unique records. After screening titles/abstracts and full texts, 10 RCTs [10–12, 17–23] were included in the meta‐analysis, comprising 1113 patients with biopsy‐confirmed MASH (fibrosis Stages F1–F4) (Figure 1). These trials were all Phase 2, double‐blind, placebo‐controlled studies conducted in North America, Europe, and Asia between 2019 and 2024. The pooled trial duration ranged from 12 to 48 weeks. The FGF21 analogues studied included efruxifermin (ALXN 1920, an Fc‐FGF21 fusion protein) in five trials, pegbelfermin (a PEGylated FGF21) in three trials, and pegozafermin (89bio′s glycoPEG‐FGF21) in two trials. Baseline characteristics were generally comparable between active and placebo groups. Overall mean age was 55.3 years, BMI 36.8 kg/m^2^, and the majority of the patients had Type 2 diabetes in most trials. Table 1 shows detailed baseline characteristics of the included studies.

**Table 1:** Baseline characteristics of the included studies.

Study name	Trial name	Country/region	Phase	Class of FGF21 analogue	Intervention dose and schedule	Follow‐up duration	Sample size, *n*	Male, *n*	Mean age	Mean BMI	T2DM, *n*	Fibrosis stage
Harrison et al. [17]	BALANCED Cohort C	United States	Phase 2a	Efruxifermin	50 mg SC once weekly versus placebo	16 weeks	30	11	59.8	37	15	F4
Harrison et al. [18]	BALANCED	United States	Phase 2a	Efruxifermin	28, 50, or 70 mg SC once weekly versus placebo	16 weeks	80	34	52.1	37.6	41	F1–F3
Noureddin et al. [12]	HARMONY	United States	Phase 2b	Efruxifermin	28 or 50 mg SC once weekly versus placebo	96 weeks	126	49	54.7	38	90	F2–F3
Akero [22]	SYMMETRY	United States, Puerto Rico, and Mexico	Phase 2b	Efruxifermin	28 or 50 mg SC once weekly versus placebo	36 weeks	181	60	60.6	NR	NR	F4
Loomba et al. (a) [11]	ENLIVEN	United States	Phase 2b	Pegozafermin	15 or 30 mg SC once weekly, or 44 mg SC every 2 weeks versus placebo	24 weeks	222	87	55.6	36.6	147	F2–F3
Loomba et al. [23]	BIO89‐100 Phase 1b/2a	United States	Phase 1b/2a	Pegozafermin	3, 9, 18, or 27 mg SC once weekly, or 18 or 36 mg SC every 2 weeks versus placebo	12 weeks	15	3	50.6	NR	4	F1–F3
Abdelmalek et al. [19]	FALCON 2	United States and Japan	Phase 2b	Pegbelfermin	10, 20, or 40 mg SC once weekly versus placebo	48 weeks	154	56	59.4	35.6	110	F4
Loomba et al. 2023 (b) [20]	FALCON 1	United States and Japan	Phase 2b	Pegbelfermin	10, 20, or 40 mg SC once weekly versus placebo	48 weeks; primary histology at Week 24	197	81	56.9	35.6	137	F3
Sanyal et al. 2019 [10]	BMS‐986036 Phase 2a	United States	Phase 2a	Pegbelfermin	10 mg SC daily or 20 mg SC once weekly versus placebo	16 weeks	75	27	50.3	35.4	28	F1–F3
Harrison et al. [21]	SYMMETRY Cohort D/GLP‐1RA combination	United States	Phase 2b	Efruxifermin	50 mg SC once weekly added to stable GLP‐1RA versus placebo/GLP‐1RA alone	12 weeks	31	NR	57	NR	31	F1–F3

Abbreviations: FGF21, fibroblast growth Factor 21; GLP‐1RA, glucagon‐like peptide‐1 receptor agonist; NR, not reported in the current extraction table; SC, subcutaneous; T2DM, Type 2 diabetes mellitus.

### 3.2. Primary Outcomes—Fibrosis Improvement Without Worsening of MASH

**Figure 2:**
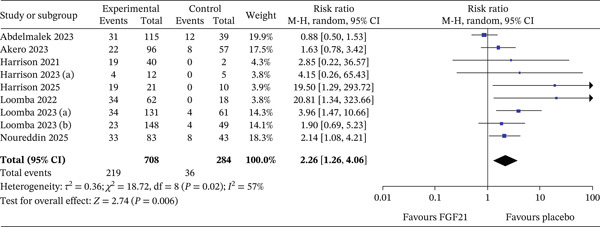
Forest plot of ≥ 1‐stage fibrosis improvement without worsening of MASH.

Figure 2 shows the forest plot of the pooled effect on fibrosis improvement. A meta‐analysis of nine RCT evaluating the efficacy of FGF21 was compared with placebo. Across nine study arms, FGF21 treatment was associated with a significantly higher likelihood of achieving the desired outcome, with a pooled RR of 2.26 (95% CI: 1.26–4.06, *p* = 0.006). This indicates that patients receiving FGF21 were more than twice as likely to achieve benefit compared with placebo. Moderate heterogeneity was observed (*I* ^2^ = 57*%*), suggesting some variability across studies, but the overall effect remained statistically significant.

### 3.3. Secondary Outcomes

The meta‐analysis of secondary efficacy outcomes revealed that FGF21 analogue therapy was associated with significant improvements in multiple hepatic and metabolic parameters compared with placebo. A relative reduction in HFF of ≥ 30% was achieved significantly more often in the treatment arm (RR: 3.14; 95% CI: 2.36–4.17). Furthermore, quantitative analysis showed a SMD of −1.59 (95% CI: −1.93 to −0.89, *p* < 0.001) regarding HFF reduction. Significant reductions were observed in ALT (SMD = −0.75, 95% CI: −0.99 to −0.48, *p* < 0.001) and AST (SMD = −0.61, 95% CI: −0.85 to −0.34, *p* < 0.001). Liver stiffness measurement (LSM) by FibroScan was also reduced (MD = −3.33, 95% CI: −4.55 to −1.87, *p* < 0.001), as were fibrosis biomarkers including Pro‐C3 (MD = −5.26, 95% CI: −7.80 to −2.50, *p* < 0.001) and ELF score (SMD = −0.91, 95% CI: −1.15 to −0.66, *p* < 0.001). In terms of lipid profile, FGF21 analogues significantly increased HDL‐C (SMD = 1.04, 95% CI: 0.77–1.44, *p* < 0.001) and decreased LDL‐C (SMD = −0.45, 95% CI: −0.68 to −0.21, *p* < 0.001). Table 2 shows the detailed analysis of secondary outcomes.

**Table 2:** Meta‐analysis of secondary efficacy outcomes.

Outcomes	Studies, *n*	FGF21 analogue, *n*	Placebo, *n*	Heterogeneity *p* value	*I* ^2^ (%)	Effect indicator	Effects model	Effect size (95% CI)	*p* value
Proportion of patients with ≥ 30% relative reduction in HFF	6	504	215	0.14	40	RR	Fixed	3.14 (2.36–4.17)	< 0.001
Relative change in HFF	6	396	170	< 0.001	75	SMD	Random	−1.59 (−1.93 to −0.89)	< 0.001
ALT	5	226	108	0.88	0	SMD	Fixed	−0.75 (−0.99 to −0.48)	< 0.001
AST	5	226	108	0.54	0	SMD	Fixed	−0.61 (−0.85 to −0.34)	< 0.001
LSM by FibroScan	4	241	122	0.91	0	MD	Fixed	−3.33 (−4.55 to −1.87)	< 0.001
Pro‐C3	5	250	124	0.03	58	MD	Random	−5.26 (−7.80 to −2.50)	< 0.001
ELF score	3	222	112	0.41	1	SMD	Fixed	−0.91 (−1.15 to −0.66)	< 0.001
HDL‐C	4	263	136	0.18	66	SMD	Random	1.04 (0.77–1.44)	< 0.001
LDL‐C	4	263	136	0.39	6	SMD	Fixed	−0.45 (−0.68 to −0.21)	< 0.001

Abbreviations: ALT, alanine aminotransferase; AST, aspartate aminotransferase; CI, confidence interval; ELF score, enhanced liver fibrosis score; FGF21, fibroblast growth factor 21; HbA1c, hemoglobin A1c; HDL‐C, high‐density lipoprotein cholesterol; HFF, hepatic fat fraction; LDL‐C, low‐density lipoprotein cholesterol; LSM, liver stiffness measurement; MD, mean difference; MRE, magnetic resonance elastography; Pro‐C3, procollagen Type III n‐terminal propeptide; RR, relative risk; SMD, standardized mean difference.

### 3.4. Safety and Adverse Events

**Figure 3:**
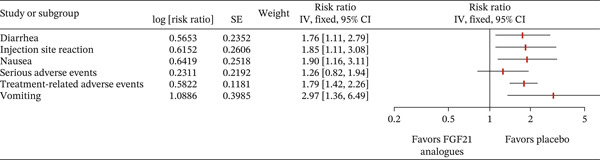
Forest plot showing analysis of adverse events [12], [15].

Treatment with FGF21 analogues was associated with a higher risk of certain adverse events compared with placebo. The risk of gastrointestinal side effects was significantly increased, including diarrhea (RR: 1.76, 95% CI: 1.11–2.79), nausea (RR: 1.90, 95% CI: 1.16–3.11), and vomiting (RR: 2.97, 95% CI: 1.36–6.49). Injection site reactions were also more common with FGF21 therapy (RR: 1.85, 95% CI: 1.11–3.08). Similarly, treatment‐related adverse events overall were significantly higher in the FGF21 group (RR: 1.79, 95% CI: 1.42–2.26). However, there was no significant difference in the incidence of serious adverse events between FGF21 analogues and placebo (RR: 1.26, 95% CI: 0.82–1.94). Figure 3 highlights the analysis of adverse events.

### 3.5. Quality Assessment

The risk of bias assessment showed that most included studies were judged to be at low risk across all five domains: randomization process (D1), deviations from intended interventions (D2), missing outcome data (D3), measurement of the outcome (D4), and selection of the reported result (D5). Two studies, Harrison et al. [18] and Sanyal et al. [10] had “some concerns” due to issues in D5 and D4, respectively. Overall, the quality of the evidence base was high, with limited concerns regarding bias (Figure S1).

### 3.6. Sensitivity Analysis

A sensitivity analysis was performed for the improvement of the fibrosis and MASH resolution outcome by excluding the studies with patients of Grade F4 fibrosis. FGF21 treatment was associated with a significantly greater measure of improvement of fibrosis and MASH resolution, with a pooled RR of 3.05 (95% CI: 1.64–5.68, *p* = 0.0004; *I* ^2^ = 27*%*) (Figure S2).

### 3.7. Certainty of Evidence

Using the GRADE framework, the certainty of evidence was assessed separately for each key outcome across the domains of risk of bias, inconsistency, indirectness, imprecision, and publication bias. Certainty was rated as moderate for ≥ 1‐stage fibrosis improvement without worsening of MASH because of moderate heterogeneity across trials (*I* ^2^ = 57*%*). Certainty was high for hepatic fat reduction, ALT, AST, and treatment‐related adverse events when estimates were precise and heterogeneity was low or acceptable. Certainty was moderate for relative change in HFF, Pro‐C3, HDL‐C, LDL‐C, LSM, ELF score, individual gastrointestinal adverse events, injection‐site reactions, and serious adverse events because of inconsistency, indirectness related to surrogate endpoints, or imprecision from wide CI. Domain‐level judgments and explanations for each outcome are provided in Table S2.

## 4. Discussion

In this meta‐analysis of randomized trials in MASH, we found that FGF21 analogue therapy significantly increases the odds of histological fibrosis improvement compared with placebo. This result was consistent across most studies and drug subclasses, with only moderate between‐study heterogeneity. Although recent reviews by Lin et al. [24] and Newsome [25] have highlighted the metabolic benefits of FGF21 analogues, our analysis provides an updated synthesis focusing specifically on the histological fibrosis endpoints using the most recent trial data. It aligns closely with previous analyses by de Oliveira et al. [26], who found an RR of 1.54 for fibrosis improvement. Our pooled estimate indicates that FGF21 analogues are indeed effective antifibrotic agents in the short term. The antifibrotic efficacy seen here is encouraging, as advanced fibrosis is the main predictor of adverse outcomes in MASH [27]. Comparable magnitude of fibrosis improvement has not been demonstrated with other drug classes; for instance, GLP‐1 receptor agonists and PPAR agonists (thiazolidinediones) have primarily shown effects on steatosis and inflammation [28, 29] with more modest fibrosis signals in trials. The observed antifibrotic benefits align with establishing biological mechanisms. Preclinical data indicate that FGF21 signaling directly modulates hepatic stellate cells, reducing collagen synthesis and dampening fibrogenic pathways [30]. Consistent with a metabolic mode of action, our analysis also confirmed robust reductions in liver fat, transaminases, and profibrotic biomarkers. Thus, the antifibrotic effect may be secondary to a reduction in lipotoxic injury. [31].

Our results can be compared with those of key individual trials. The Phase 2b ENLIVEN trial of pegozafermin [11] reported fibrosis improvement in 22%–27% of treated patients versus 7% on placebo, roughly a 3‐fold difference; this trial alone contributed strongly to our pooled estimate. Similarly, Harrison et al. [18] showed that efruxifermin markedly reduced liver fat and improved histological features, although the relatively small sample size left limited power for fibrosis endpoints. In the FALCON‐1 trial of pegbelfermin [20], the highest dose group met the primary endpoint of fibrosis improvement (19% vs. 4% in the placebo group) [10]. Although FALCON‐2 [19] and other smaller studies also hinted at a benefit, the sample sizes were limited. In aggregate, our meta synthesizes these discrete findings into a more precise estimate, reinforcing that multiple FGF21 analogues exert a similar effect. Notably, subgroup analysis from de Oliveira et al. [26] suggests that the antifibrotic effect may be confined to noncirrhotic patients (Fibrosis F1–F3); this is plausible given the more dynamic nature of fibrogenesis before cirrhosis is established.

We observed only mild to moderate heterogeneity. Variability could arise from differences in patient populations (e.g., baseline fibrosis stage and diabetes prevalence), trial duration (16–48 weeks), and specific drug/dosing regimens. Some heterogeneity may also reflect the inherently subjective nature of histological endpoints; indeed, trials often report ranges of response. Importantly, the heterogeneity did not meaningfully alter the overall conclusions.

Safety and tolerability are critical for any chronic MASH therapy. Our pooled data showed no increase in serious adverse events with FGF21 analogues. This is reassuring and consistent with earlier meta‐analyses. [26] The most frequent side effects were gastrointestinal—principally nausea and diarrhea—occurring in a minority of patients. These are not unexpected, given the effects on gut hormones and motility. They were generally transient and manageable and did not lead to many discontinuations. For perspective, the only approved MASH drug (resmetirom) also lists diarrhea and nausea as common side effects. [32] Injection‐site erythema was occasionally noted with weekly dosing, but similarly mild. Overall, the safety profile of FGF21 analogues appears comparable to other metabolic agents (GLP‐1 RAs and PPAR agonists), all of which cause predominant GI symptoms. [24].

Several limitations should be acknowledged. First, all included studies were relatively short‐term, Phase 2 trials. Histological changes in liver fibrosis typically evolve slowly, and we cannot infer the durability of the effect beyond the trial periods. Long‐term data will be needed to confirm sustained fibrosis regression and to evaluate clinical outcomes like cirrhosis development and liver‐related events. Second, although the total number of patients was substantial for a niche field, individual trials were modest in size. Third, patient populations in trials were predominantly middle‐aged, obese, and diabetic individuals. Our results may not generalize to all MASH patients who have low BMI or those with decompensated cirrhosis. Fourth, we could not assess publication bias formally with funnel plots due to fewer than 10 trials per analysis; unpublished negative trials cannot be excluded. Finally, we included studies labeled as MASH (the older nomenclature) as proxies for MASH, but the pathophysiology is essentially identical. We maintained consistency by describing outcomes in the MASH framework.

Clinically, our findings have several implications. They suggest that FGF21 analogues could emerge as effective antifibrotic therapies in MASH. Given the lack of other approved antifibrotic drugs, a risk reduction of 2.25 is meaningful. If confirmed in larger Phase 3 trials (some of which are already planned or underway), FGF21 analogues might be used in patients with moderate‐to‐severe MASH (especially F1–F3 fibrosis) to halt or reverse disease progression. Combination therapy is another consideration: Several preclinical and early human studies are exploring FGF21 analogues with GLP‐1 agonists or SGLT2 inhibitors, which could yield additive metabolic benefits. In addition, monitoring of fibrosis biomarkers (e.g. Pro‐C3 and ELF) may help identify responders early. For future research, longer follow‐up is crucial. Trials should assess hard clinical endpoints (progression to cirrhosis and cardiovascular outcomes) and perform subgroup analyses (e.g., diabetic vs. nondiabetic). It will also be important to determine whether the fibrosis benefit plateaus or continues with prolonged therapy, and whether the drug effect diminishes after cessation.

## 5. Conclusion

This meta‐analysis demonstrates that FGF21 analogue therapy significantly improves liver fibrosis in patients with MASH, with a favorable safety profile. These agents hold promise as a novel class of treatment for steatohepatitis‐related fibrosis. Confirmation in large, long‐term trials is warranted to establish their role in routine practice.

## Author Contributions

Muhammad Naqash: conceptualization, data curation, supervision, and writing—review and editing; Abdullah Tariq: writing—original draft and writing—review and editing; Shazma Shayan: writing—original draft and writing—review and editing; Sadaf Manzoor Sargani: writing—original draft and writing—review and editing; Muhammad Asad Raza: resources, writing—original draft, and writing—review and editing; Nasar Nasrullah Khan: formal analysis and writing—review and editing; Aiman Balouch: writing—original draft and writing—review and editing; Rana Muhammad Usama: writing—original draft and writing—review and editing; Khabab Abbasher Hussien Mohamed Ahmed: writing—original draft and writing—review and editing.

## Funding

No funding was received for this manuscript.

## Ethics Statement

The study protocol was registered with the International PROSPERO Registry (CRD420251017463). This was a systematic review and not a clinical trial.

## Consent

The authors have nothing to report.

## Conflicts of Interest

The authors declare no conflicts of interest.

## Supporting information


**Supporting Information** Additional supporting information can be found online in the Supporting Information section. Table S1: Detailed search strategy used in each database. Table S2: GRADE certainty assessment by outcome. Figure S1: Quality assessment of the included studies. Figure S2: Sensitivity analysis for the primary outcome by excluding Stage F4 fibrosis studies.

## Data Availability

The data that supports the findings of this manuscript were made all available within this manuscript or the Supporting Information.
