# Supplementary material for: Fibroblast Growth Factor 21 Analogues Improve Fibrosis in Metabolic Dysfunction–Associated Steatohepatitis: An Updated Systematic Review and Meta‐Analysis
Source: Int J Hepatol. 2026 Jun 3;2026:7391450. doi: 10.1155/ijh/7391450 (PMC13239190; doi:10.1155/ijh/7391450)
Supplement: Supplementary file 1 — Supporting Information Additional supporting information can be found online in the Supporting Information section. Table S1: Detailed search strategy used in each database. Table S2: GRADE certainty assessment by outcome. Figure S1: Quality assessment of the included studies. Figure S2: Sensitivity analysis for the primary outcome by excluding Stage F4 fibrosis studies. [file IJH-2026-7391450-s001.docx]

**Supplementary Material**

| PubMed | ("fibroblast growth factor 21"[Supplementary Concept] OR "fibroblast growth factor 21"[All Fields] OR "fgf21"[All Fields]) AND ("MASLD"[All Fields] OR ("non alcoholic fatty liver disease"[MeSH Terms] OR ("non alcoholic"[All Fields] AND "fatty"[All Fields] AND "liver"[All Fields] AND "disease"[All Fields]) OR "non alcoholic fatty liver disease"[All Fields] OR "nash"[All Fields])) |
| --- | --- |
| Cochrane Library | (FGF21) AND (MASLD OR NASH OR non-alcoholic steatohepatitis) |
| Scopus | (FGF21) AND (MASLD OR NASH OR non-alcoholic steatohepatitis) |

Table S1. Detailed search strategy used in each database.


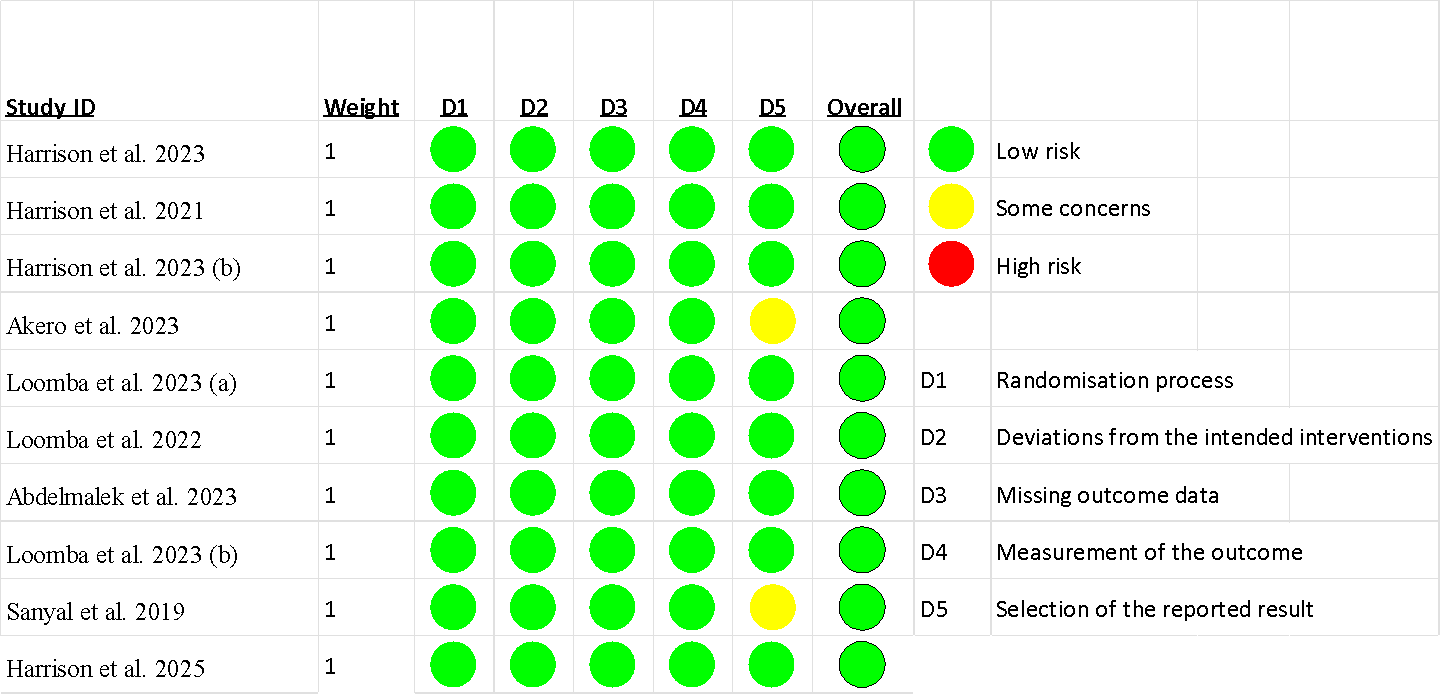


Figure S1. Quality assessment of the included studies.


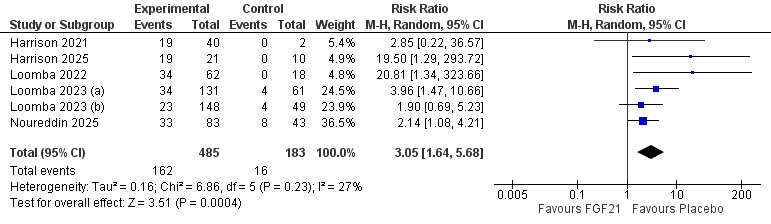


Figure S2. Sensitivity analysis for the primary outcome by excluding stage F4 fibrosis studies.

Table S2. GRADE certainty assessment by outcome

| **Outcome** | **Studies, n** | **Participants, n** | **Effect estimate** | **Risk of bias** | **Inconsistency** | **Indirectness** | **Imprecision** | **Publication bias** | **Overall certainty** | **Explanation** |
| --- | --- | --- | --- | --- | --- | --- | --- | --- | --- | --- |
| ≥1-stage fibrosis improvement without worsening of MASH | 9 | 977 | RR 2.26 (95% CI 1.26 to 4.06) | Some concerns | Serious | Not serious | Not serious | Not assessable | Moderate | Downgraded one level for inconsistency due to moderate heterogeneity (I² = 57%). Risk-of-bias concerns were present in selected domains but were not judged sufficient for an additional downgrade. |
| ≥30% relative reduction in hepatic fat fraction (HFF) | 6 | 719 | RR 3.14 (95% CI 2.36 to 4.17) | Not serious | Not serious | Not serious | Not serious | Not assessable | High | No serious concerns for risk of bias, inconsistency, indirectness, or imprecision; heterogeneity was not substantial (I² = 40%). |
| Relative change in HFF | 6 | 566 | SMD -1.59 (95% CI -1.93 to -0.89) | Not serious | Serious | Not serious | Not serious | Not assessable | Moderate | Downgraded one level for inconsistency due to substantial heterogeneity (I² = 75%). |
| ALT | 5 | 334 | SMD -0.75 (95% CI -0.99 to -0.48) | Not serious | Not serious | Not serious | Not serious | Not assessable | High | No serious concerns; heterogeneity was absent (I² = 0%) and the estimate was precise. |
| AST | 5 | 334 | SMD -0.61 (95% CI -0.85 to -0.34) | Not serious | Not serious | Not serious | Not serious | Not assessable | High | No serious concerns; heterogeneity was absent (I² = 0%) and the estimate was precise. |
| LSM by FibroScan | 4 | 363 | MD -3.33 (95% CI -4.55 to -1.87) | Not serious | Not serious | Serious | Not serious | Not assessable | Moderate | Downgraded one level for indirectness because liver stiffness measurement is a surrogate marker rather than a direct histological or patient-important clinical endpoint. |
| Pro-C3 | 5 | 374 | MD -5.26 (95% CI -7.80 to -2.50) | Not serious | Serious | Serious | Not serious | Not assessable | Moderate | Downgraded one level overall because of inconsistency (I² = 58%) and the surrogate nature of the biomarker; no additional downgrade was applied to avoid double counting related limitations. |
| ELF score | 3 | 334 | SMD -0.91 (95% CI -1.15 to -0.66) | Not serious | Not serious | Serious | Not serious | Not assessable | Moderate | Downgraded one level for indirectness because ELF score is a surrogate fibrosis biomarker. |
| HDL-C | 4 | 399 | SMD 1.04 (95% CI 0.77 to 1.44) | Not serious | Serious | Serious | Not serious | Not assessable | Moderate | Downgraded one level overall because of inconsistency (I² = 66%) and the surrogate metabolic nature of the endpoint; no additional downgrade was applied. |
| LDL-C | 4 | 399 | SMD -0.45 (95% CI -0.68 to -0.21) | Not serious | Not serious | Serious | Not serious | Not assessable | Moderate | Downgraded one level for indirectness because LDL-C is a surrogate metabolic endpoint. |
| Diarrhea | 10 | 1173 | RR 1.76 (95% CI 1.11 to 2.79) | Not serious | Not serious | Not serious | Serious | Not assessable | Moderate | Downgraded one level for imprecision because the confidence interval was relatively wide despite statistical significance. |
| Nausea | 10 | 1173 | RR 1.90 (95% CI 1.16 to 3.11) | Not serious | Not serious | Not serious | Serious | Not assessable | Moderate | Downgraded one level for imprecision because the confidence interval was wide. |
| Vomiting | 10 | 1173 | RR 2.97 (95% CI 1.36 to 6.49) | Not serious | Not serious | Not serious | Serious | Not assessable | Moderate | Downgraded one level for imprecision because the confidence interval was wide and event counts were likely low. |
| Injection-site reactions | 7 | 786 | RR 1.85 (95% CI 1.11 to 3.08) | Not serious | Not serious | Not serious | Serious | Not assessable | Moderate | Downgraded one level for imprecision because the confidence interval was wide. |
| Treatment-related adverse events | 7 | 786 | RR 1.79 (95% CI 1.42 to 2.26) | Not serious | Not serious | Not serious | Not serious | Not assessable | High | No serious concerns; the estimate was precise and directionally consistent. |
| Serious adverse events | 10 | 1173 | RR 1.26 (95% CI 0.82 to 1.94) | Not serious | Not serious | Not serious | Serious | Not assessable | Moderate | Downgraded one level for imprecision because the confidence interval crossed the null effect. |

Abbreviations: ALT, alanine aminotransferase; AST, aspartate aminotransferase; CI, confidence interval; ELF, enhanced liver fibrosis; GRADE, Grading of Recommendations Assessment, Development and Evaluation; HDL-C, high-density lipoprotein cholesterol; HFF, hepatic fat fraction; LDL-C, low-density lipoprotein cholesterol; LSM, liver stiffness measurement; MASH, metabolic dysfunction-associated steatohepatitis; MD, mean difference; Pro-C3, procollagen type III N-terminal propeptide; RR, risk ratio; SMD, standardized mean difference.

*Note: Publication bias was considered not assessable because the number of studies contributing to most outcome-specific analyses was small. Serious adverse events were completed using the same study and participant count as the overall safety set; verify against the final RevMan file if the serious-adverse-event analysis used fewer studies.*
